# Supplementary material for: Twelve phosphomimetic mutations induce the assembly of recombinant full-length human tau into paired helical filaments
Source: eLife. 2026 May 20;14:RP104778. doi: 10.7554/eLife.104778 (PMC13189620; doi:10.7554/eLife.104778)
Supplement: Figure 1—figure supplement 1—source data 1. [file elife-104778-fig1-figsupp1-data1.pdf]

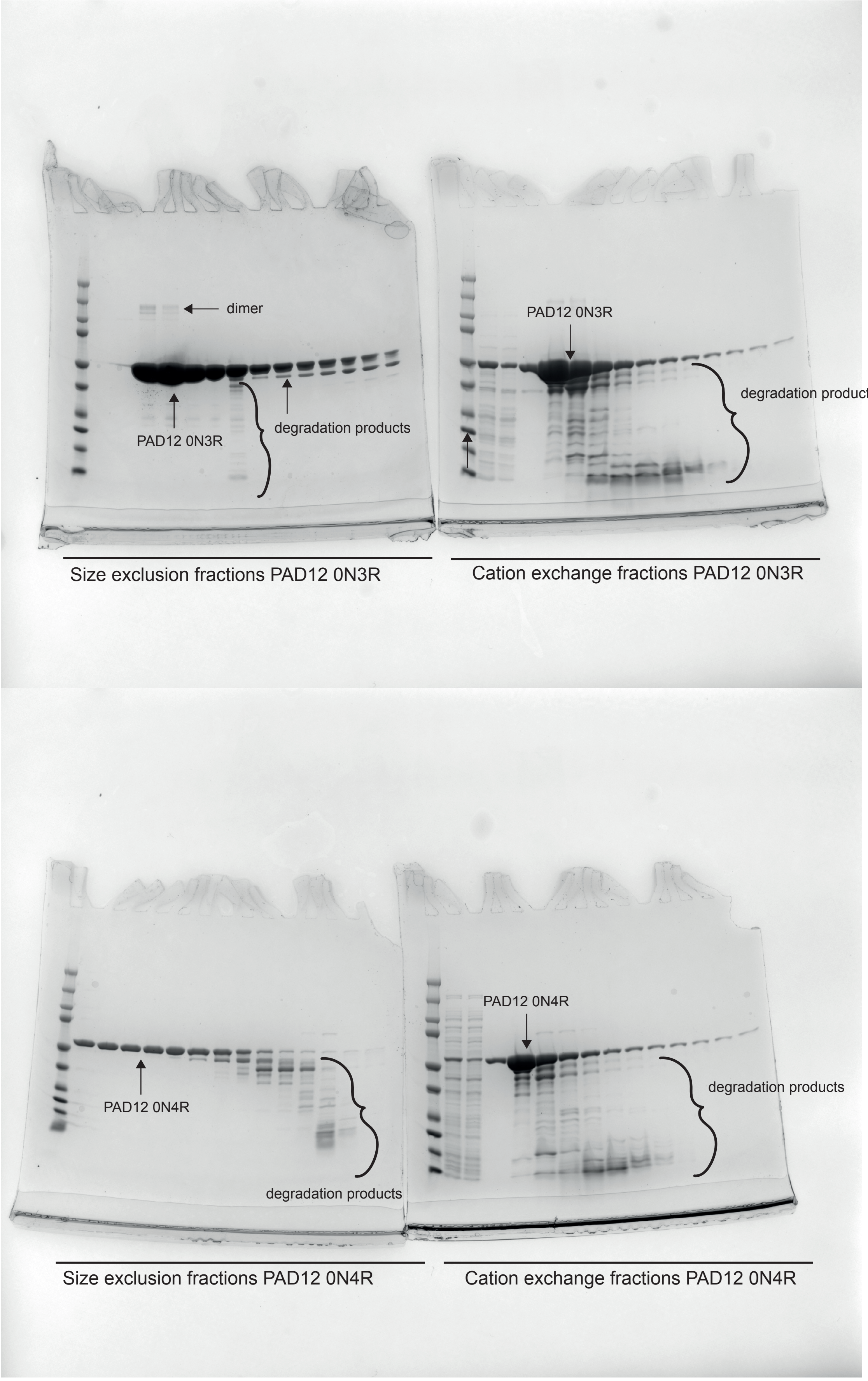

Source data for Figure 1 - Figure Supplement 1

Scanned gels for panels C & B (top left & right) and panels F & E (bottom left and right).
