# Supplementary figures and images for: Twelve phosphomimetic mutations induce the assembly of recombinant full-length human tau into paired helical filaments
Source: eLife. 2026 May 20;14:RP104778. doi: 10.7554/eLife.104778 (PMC13189620; doi:10.7554/eLife.104778)

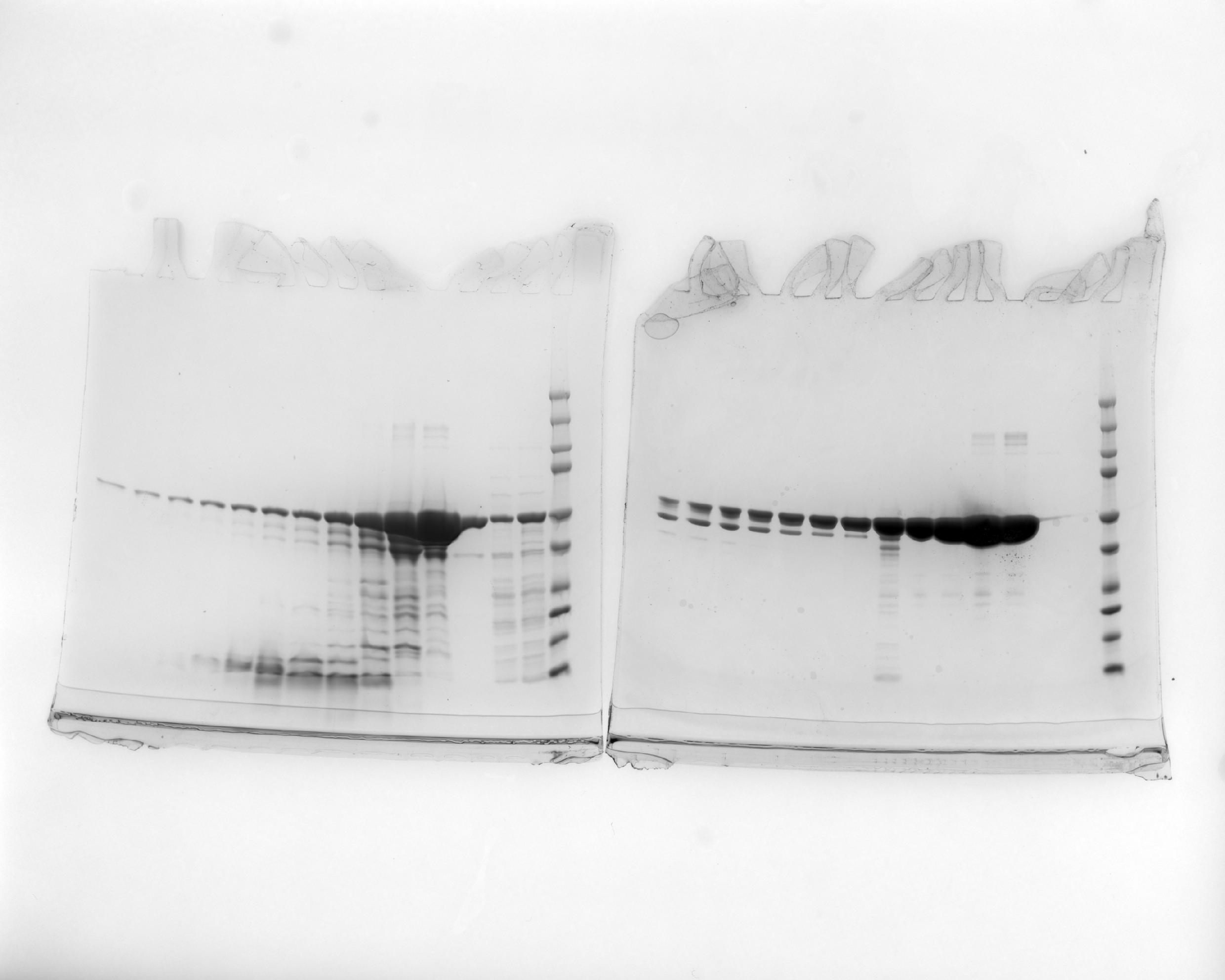

Supplement: Figure 1—figure supplement 1—source data 2. [file elife-104778-fig1-figsupp1-data2.zip › Figure1-supplement1-raw-images/Figure1-supplement1BC-source.jpg]

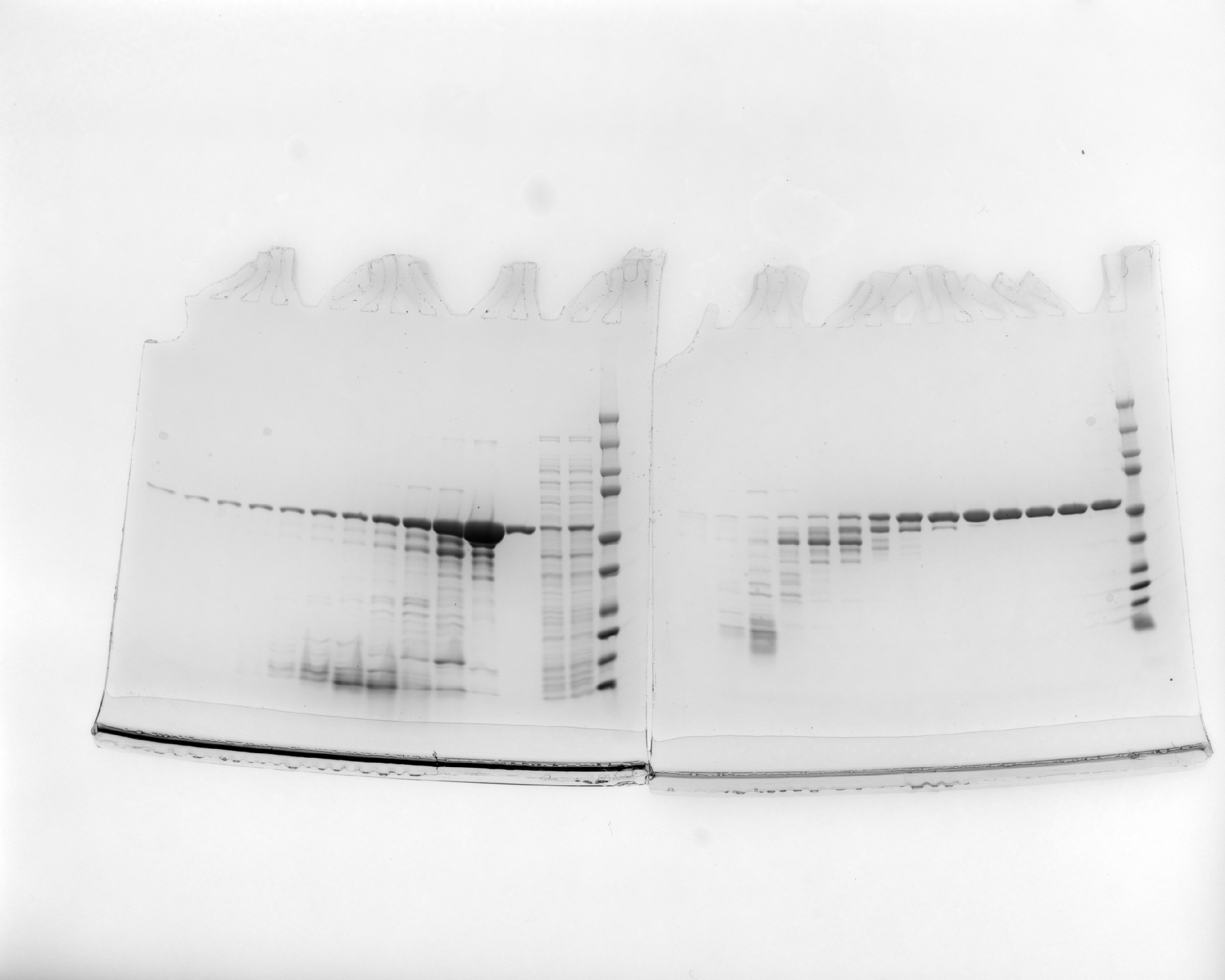

Supplement: Figure 1—figure supplement 1—source data 2. [file elife-104778-fig1-figsupp1-data2.zip › Figure1-supplement1-raw-images/Figure1-supplement1EF-source.jpg]
